# Ludwig's angina: An alarming radiology challenge^☆^

**DOI:** 10.1016/j.radcr.2022.05.085

**Published:** 2022-06-24

**Authors:** Feredy Gunawan, Widiana Ferriastuti

**Affiliations:** Department of Radiology, Faculty of Medicine Universitas Airlangga - Dr. Soetomo Academic General Hospital, Jalan Mayjen. Prof. Dr. Moestopo 47Surabaya - 60131, Surabaya, Indonesia

**Keywords:** Ludwig's angina, CT scan, Cellulitis, Abscess

## Abstract

Ludwig's angina is a cellulitis that affects the submandibular, sublingual, and submental regions, fast-spreading along the fascial plane. Ludwig's angina has been classified as a fast-moving, frequently fatal gangrenous cellulitis or necrotizing fasciitis of the neck and mouth floor over the previous 2 centuries. A 60-years old male patient came with a chief complaint of swelling and pain in the neck that radiated to the chest with fever, headache, nausea, and history of toothache, poor oral hygiene, and diabetes mellitus. Ludwig's angina with mediastinitis complication was established by a computed tomography (CT) scan, which was promptly treated and he recovered completely. Imaging is used as one of the tools to help physicians diagnose Ludwig's angina. A trained, multidisciplinary team is required for the treatment of Ludwig's angina because it involves surgical and antibiotic treatment and also resuscitation. A more accurate and timely diagnosis can lead to a better prognosis. In an emergency, a CT scan may be the best imaging choice, although magnetic resonance imaging is superior to a CT scan since it evaluates soft tissue and compartment involvement better.

## Introduction

Ludwig's angina is an uncommon infection that causes deadly submandibular gangrenous cellulitis on both sides of the mouth. The sickness was initially described in 1836 by a German physician named Wilhelm Fredrich Von Ludwig. Ludwig's angina is caused by streptococci and/or anaerobic bacteria invading the submandibular, sublingual, and submental regions, with the possibility of spreading to the mediastinum. Common clinical findings include odynophagia, edema, and trismus. This disease could be life-threatening because of airway obstruction or septic shock, which requires specific airway resuscitation and treatment. Ludwig's angina could result in a 50% mortality rate. This number could be decreased if the adequate treatment that involves surgical and antibiotic treatment and also resuscitation is given to the patient. Ludwig's angina can be caused by an infection in the mouth, penetrating trauma to the mouth floor, osteomyelitis or a jaw fracture, otitis media, tongue piercing, or sialolithiasis of the submandibular gland [1,2].

## Case report

A 60-year-old patient presented to our emergency room after being transferred from another hospital with a chief complaint of neck swelling for 2 weeks prior to admission. He also had swelling with tenderness and warmth. Two days before the patient was admitted, the pain and warmth radiated to the chest and the patient complained change of voice and difficulty opening the mouth and eating. Prior to admission, the patient had been sick for 5 days with a slight temperature, headache, and nausea. The patient had been suffering from throat soreness for a week before to admission. Neither cough nor shortness of breath was felt.

Three months before admission, the patient underwent a teeth extraction procedure because of recurring toothache. The patient previously only consumed pain-relieving drugs to treat the toothache. The patient had a history of poor oral hygiene. He also had substantial jaw pain 2 weeks before being sent to the hospital. He had been diagnosed with type 2 diabetes 2 days before being referred to our hospital.

The patient was awake and alert when admitted but in poor health. Blood pressure was 107/68 mm Hg, heart rate was 78 beats/minute, respiratory rate was 20 times/min, the temperature was 37°C, and peripheral oxygen saturation was 98 percent with 10 lpm of O2 using a simple mask. A muffled voice was found. Trismus with 2 fingers width, standing secretion, and many dental cavities were discovered during a mouth examination. A neck examination revealed solid, hyperemic submandibular edema with fluctuation and crepitation. A solid, hyperemic edema was also found on the right neck, however, no fluctuation was found. There was also edema in the thorax area with hyperemia above the clavicula. A nose examination revealed no abnormality.

**Fig. 1:**
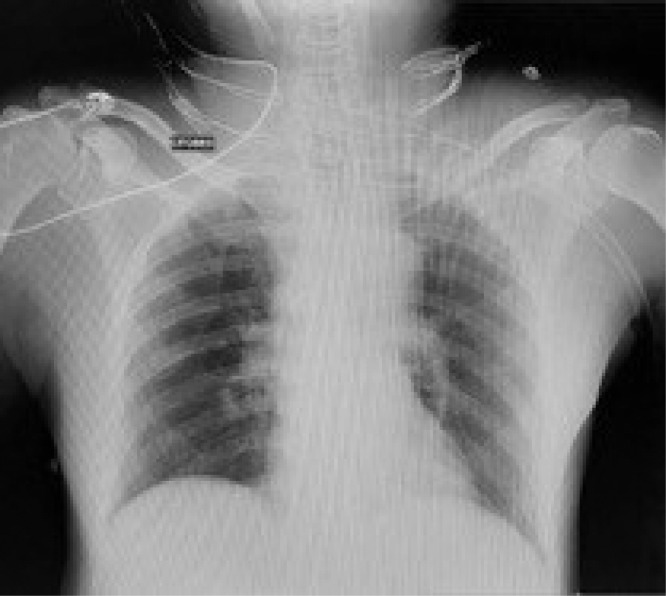
Chest x-ray.

**Fig. 2:**
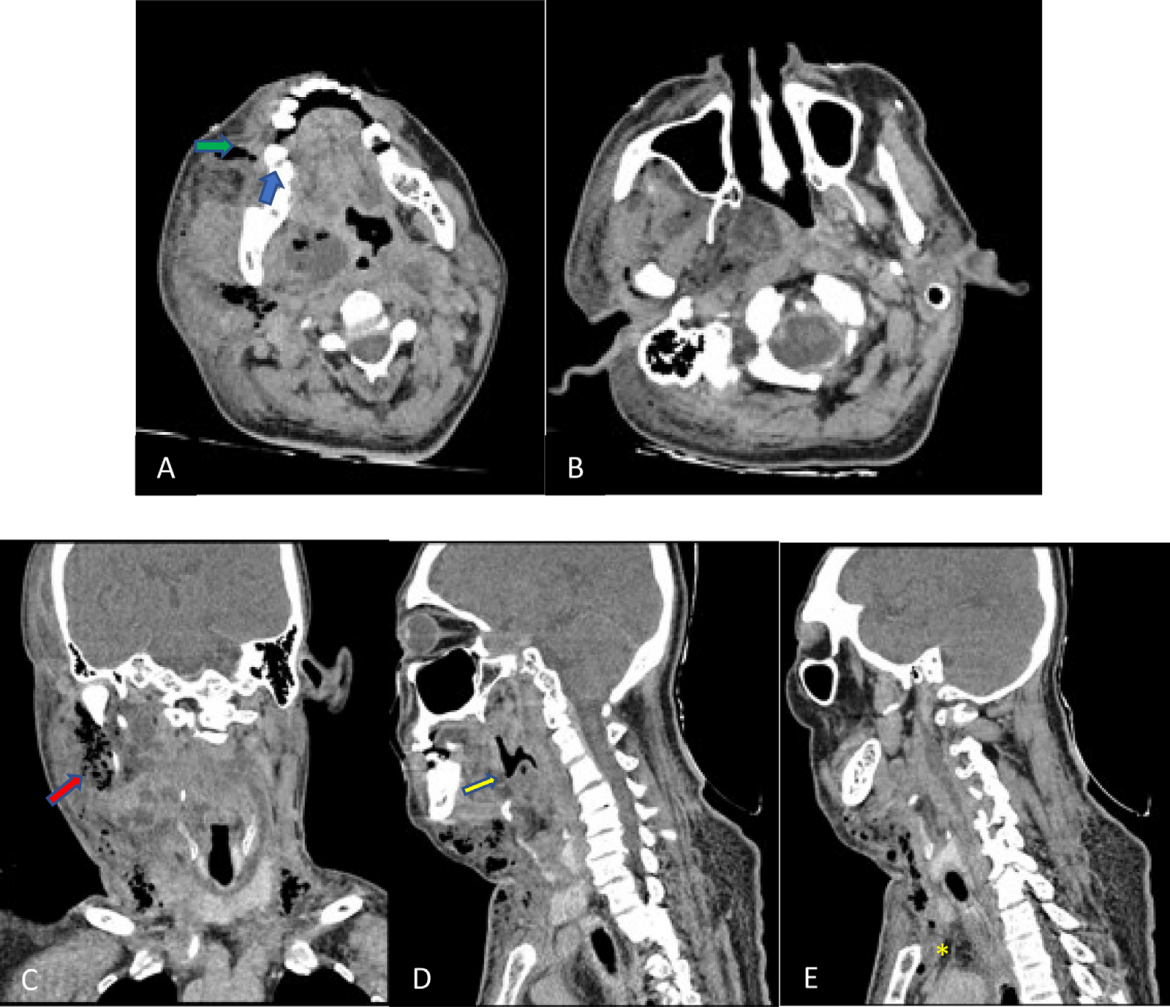
A contrast CT scan of the neck's soft tissue. (A) Periapical abscess of the right first molar (green arrow, axial image) with soft tissue swelling around it that extended to the right sublingual space (blue arrow, axial image), (B) The abscess extended to the buccal space, cutaneous-subcutaneous of temporomandibular, right and left parapharyngeal space, and danger space. (C) Right stylomandibular canal (red arrow, coronal image) (D) The abscess caused airway obstruction with a diameter ±2 mm (yellow arrow, sagittal image) (E) The abscess also extended to mediastinal caused mediastinitis (yellow asterix, sagittal image).

Laboratory examination results were as follow: hemoglobin 10.8 (normal value: 13.3-16.6 g/dL), white blood cells 21.31 × 10^3^ (normal value: 3.37-10.0 × 10^3^/µL), Albumin level 2.84 (normal value 3.4- 5.0 g/dL), blood urea nitrogen level 53 (normal value: 7-18 mg/dL), SGOT 70 (normal value: 5-34 U/L), and SGPT 69 (normal value: 0-55 U/L). A cervical x-ray was already performed from the previous hospital with findings suggesting a right submandibular abscess with paracervical muscle spasm. In our emergency room, a chest x-ray was performed with normal findings (Fig. 1). A computed tomography (CT) scan (Fig. 2) with intravenous (IV) contrast revealed a periapical abscess of the right first molar with soft tissue swelling around it. The abscess then spread to the right sublingual space, buccal space, cutaneous-subcutaneous temporomandibular area, right and left parapharyngeal space, danger space, oropharynx, hypopharynx, epiglottis space, right and left prevertebral space, until retrosternal space at the level of second thoracic vertebrae, which reached the cutaneous-subcutaneous and mediastinal fat at the front side of vertebrae. The abscess also caused airway obstruction at the level of the first-fifth cervical vertebrae, with the narrowest diameter ± 0.2 cm.

Based on the examination results, the patient was diagnosed with submandibular abscess + suspected right parapharyngeal abscess + suspected mediastinitis abscess + diabetes mellitus type 2 + hypoalbumin. The patient received ringer lactate infusion 1500 mL/24 hours, IV ceftriaxone 1 g/12 hours (third day), IV metronidazole 500 mg/8 hours, and IV metamizole 1 g/8 hours (third day). The patient was intubated to secure airway patency and was planned for emergency surgery to drain the abscess.

Pus was discovered during the incision, but no further findings were discovered. The pus was drained by an otolaryngologist, and the patient was transported to the intensive care unit. The patient was discharged from the hospital several days following the surgery, having fully recovered.

## Discussion

Ludwig's angina is life-threatening cellulitis that progresses rapidly along the fascial plane. In this case report, our patient developed Ludwig's angina from untreated dental caries and poor oral hygiene. The right first molar is the most often infected tooth. Other potential sources of infection that have already been reported include sialadenitis, submandibular gland sialolithiasis, peritonsillar abscess, mandibular fracture, mandibular osteomyelitis, penetrating trauma of the mouth floor, and tongue piercing [3].

Diabetes, poor oral hygiene, malnutrition, obesity, alcoholism, and other conditions are all major risk factors. The usage of nonsteroidal anti-inflammatory drugs and/or self-medication may have led to the development of this oropharyngeal infection [2]. Diabetes mellitus and poor dental hygiene were noted by the patient in this case.

The patient's airway was secured with intubation, given broad-spectrum antibiotics, and was planned for emergency surgery to drain the abscess. Cellulitis of the submandibular area can migrate to the parapharyngeal space, then the retropharyngeal space, and lastly the superior mediastinum. Ludwig's angina is a life-threatening condition because it could cause massive soft tissue edema and tongue movement towards the posterior side, which leads to airway narrowing. Airway management is the main goal of treatment. Airway maintenance, whether with endotracheal intubation (if possible) or with tracheostomy or cricothyroidotomy, is required and followed by antibiotic and surgical treatment. The majority of Ludwig's angina patients are polymicrobial, containing both positive and negative gram bacteria as well as anaerobic bacteria. *Streptococcus viridans, Staphylococcus aureus, Neisseria species, Haemophilus species, Bacteroides species, non-A-streptococcus, Streptococcus pyogenes, Fusobacterium, Prevotella,* and *Eikenella*, in that order, are the most often isolated organisms [3].

Our patient exhibited tooth discomfort, dysphagia, odynophagia, and a muffled voice in this case report. The most common signs and symptoms of Ludwig's angina include dental and oral discomfort, dysphagia and odynophagia, sore throat, otalgia, respiratory distress, and a change in voice. Swelling of the neck, trismus, halitosis, sialorrhea, gingivitis, or a muffled voice may be discovered during an examination. Other physical findings that are reported in kinds of literature include “double tongue sign”, which is mouth floor elevation that is caused by submandibular space edema. During palpation, cervical adenopathy and “woody” characteristic of the mouth floor [4].

Our patient had a chest x-ray and a contrast-enhanced CT scan, which revealed a periapical abscess of the right first molar with soft tissue swelling around it. The abscess's extent was detected via a contrast-enhanced CT scan. Airway obstruction was also noted from the imaging. Ludwig's angina requires a radiological study to diagnose. Ludwig's angina can be diagnosed with a CT scan or an magnetic resonance imaging by specificity and sensitivity. It is crucial for imaging modality to determine the airway patency of the patient [5].

## Conclusion

Ludwig's angina is a type of cellulitis that produces airway occlusion and is rare, progressive, and potentially lethal. The progression of the condition is influenced by oral hygiene and dental cavities. Imaging studies in an emergency setting such as a CT scan might help physicians with its diagnosis, even though magnetic resonance imaging is better at evaluating soft tissue.

## Informed consent revised

Informed consent obtained for publication of a case report.

Written informed consent was obtained from the patient for the publication of this case report.
